# Expanded span of control, leadership and management performance, work-related stress, and job satisfaction among first-line managers: A repeated cross-sectional study

**DOI:** 10.1177/10519815251326470

**Published:** 2025-04-03

**Authors:** Jonas Svanström, Bernice Skytt, Maria Lindberg, Magnus Lindberg

**Affiliations:** 1 Department of Caring Sciences, University of Gävle, Gävle, Sweden; 2 Department of Public Health and Caring Sciences, Uppsala University, Uppsala, Sweden; 3 Centre for Research and Development, Region Gävleborg/Uppsala University, Gävle, Sweden

**Keywords:** first-line manager, job satisfaction, leadership, management, span of control, work-related stress

## Abstract

**Background:**

First-line healthcare managers navigate complex organizational demands to ensure a good work environment and quality care. Key factors such as expanded span of control, leadership and management performance, and work-related stress significantly influence their job satisfaction. However, how these factors evolve over time in organizational settings remains unclear.

**Objective:**

To examine expanded span of control, leadership and management performance, work-related stress, and job satisfaction among first-line healthcare managers and assess whether the relationships between these variables remained stable over time.

**Methods:**

A repeated cross-sectional design was used to collect annual data from 2020 to 2023 among first-line managers in a Swedish healthcare organization. The Ottawa Hospital Span of Control tool, the Leadership and Management Inventory, the Health & Safety Executive stress tool, and a single-item job satisfaction measure were used to collect data. Kruskal-Wallis tests and Spearman correlation analysis were performed.

**Results:**

An expanded span of control negatively impacted job satisfaction in certain years. Leadership and management performance showed a positive but inconsistent association with job satisfaction. Factors in work-related stress, particularly high demands and strained relationships, consistently had a negative relationship with job satisfaction, whereas control and support positively contributed to greater job satisfaction.

**Conclusions:**

Work-related stress, driven by high demands and poor relationships, significantly decreases job satisfaction. Leadership and management performance influence satisfaction, but inconsistently. Reducing workload and improving support structures can enhance job satisfaction and managerial effectiveness.

## Introduction

First-line managers in healthcare operate in a dynamic and ever-evolving environment, shaped by factors such as political reforms, organizational demands, and the need to implement evidence-based practices. This can lead to uncertainty, heavier workloads, and tight deadlines.^1,2^ Additionally, frequent reforms contribute to significant organizational pressure, straining both staff and the organization's overall functioning.^3^

In the aforementioned context, first-line managers balance the competing demands of staff and upper management to ensure that organizational goals and political demands are met.^4^ Their responsibilities require first-line managers to demonstrate both leadership and management. As defined by Alghatani,^5^ leadership involves influencing and guiding staff towards a shared vision, requiring qualities such as integrity, decisiveness, trust, and effective communication. Management focuses on directing and controlling operations through administrative and supervisory roles, ensuring staff development, conflict resolution, and maintenance of ethical standards.^5^ First-line managers are expected to handle both leadership and management adeptly, adapting higher management directives to suit their unit's needs, fostering a good work environment, and ensuring high-quality patient care.^6^ Operating in such a dynamic environment requires first-line managers to make quick decisions, manage heavy workloads, and ensure effective team collaboration under pressure.^6^ Consequently, the role requires skills in resource management, communication, and leadership. A skilled first-line manager ensures that the unit functions efficiently and that staff are supported in delivering optimal care, while also fostering teamwork.^7^

The workload of first-line managers is often described using the concept of “span of control,” which originally referred to the number of employes overseen by a manager.^8^ Recent research has expanded the concept of span of control to include factors such as the number of employes reporting directly to a manager, employe characteristics, required supervision level, work complexity, and the manager's capabilities.^9,10^ Here, this is referred to as “expanded span of control.” These broader considerations highlight that the first-line manager's role extends far beyond just the number of employes, for instance significantly shaping nurses’ experiences of their work environment. The extent of the span of control, combined with communication practices, plays a crucial role in promoting job satisfaction within nursing units. However, as the expanded span of control grows, managers often find it harder to maintain close connections with their staff, which can undermine their ability to provide effective leadership and support.^11^

Research has shown that work-related stress is a common issue among first-line managers, with many experiencing moderate to high levels of stress.^12^ An integrative review by Labrague et al.^13^ identifies several interconnected factors contributing to this stress, such as high job demands, resource limitations, and financial management responsibilities. These factors are further compounded by heavy workloads, inadequate staffing, and the constant need to balance organizational objectives with staff needs. The review by Labrague et al.^13^ revealed that limited decision-making authority and unclear role boundaries contribute to stress, with financial responsibilities, especially when lacking proper training, intensifying the strain. The workload for first-line managers increased during the COVID-19 pandemic, exacerbating existing challenges. For example, during the pandemic, healthcare managers reported significant increases in dissatisfaction with role clarity, decision-making authority, and work-life balance.^14^

High stress levels are consistently linked to lower job satisfaction, a more negative perception of the work environment, and an increased intention to leave one's role.^15,16^ Various factors significantly influence job satisfaction among first-line managers. A systematic review by Penconek et al.^17^ highlighted the multifaceted nature of job satisfaction among first-line managers, showing that a wide range of factors shaped the overall sense of fulfillment. These can be divided into three key areas: job characteristics, organizational factors, and personal traits. At the job level, aspects like autonomy, authority in decision-making, and supportive relationships with colleagues and superiors positively impacted job satisfaction. However, when first-line managers face overwhelming workloads, role overload, and insufficient support, their job satisfaction tends to decrease.^17^

Despite the extensive changes in the healthcare sector and the significant role that first-line managers play in leading their units, it remains unclear how their job satisfaction, work-related stress, leadership and management performance, and span of control are impacted over time. First-line managers work in a dynamic environment that requires balancing multiple demands. Understanding these dynamics is important for improving first-line managers’ well-being and effectiveness. Inspired by the Job Demand Resourses (JD-R) model,^18^ we developed three hypotheses in this study. The first hypothesis (H1) was that there would be no differences in job satisfaction, perceived management support, peer support, control, demands, relationships, role clarity, attitudes towards change, leadership and management performance among first-line managers when cross-sectional data collected annually from 2020 to 2023 were compared. In hypothesis 2 (H2), we expected to find a positive correlation between job satisfaction and leadership and management performance, managerial support, peer support, control, role, and change. Conversely, we expected a negative correlation between job satisfaction and demands, relationships, and expanded span of control when cross-sectional data collected annually from 2020 to 2023 were analyzed. In the third and final hypothesis (H3), we assumed that the calculated correlations (in H2) would demonstrate comparable strength over time (2020–2023).

## Methods

### Design, sampling and data collection procedure

This study employed a repeated cross-sectional design, with data collected annually during the autumn months of 2020–2023. The study was conducted in a region of Sweden with around 300,000 residents and about 6500 employes in the healthcare organization. This setting included first-line managers working in primary, pre-hospital, and inpatient care.

We used a convenience sampling procedure of all first-line managers employed in healthcare sectors within the region during the data collection periods, no other eligible criterion was used. The sample size was limited by the number of eligible first-line managers working in the healthcare sector during each data collection period. Although response rates varied across the years, the sample size was sufficient for the statistical analyses.^19^ Further details on the sample size for each year can be found in Table 1. About a month before the annual data collection, we received a list of current first-line managers from the region's human resource department. We emailed the intended participants detailed written information about the study and a link to the electronic survey. Non-respondents received two email reminders in an effort to improve the response rates.

**Table 1.** Characteristics of participants.

Variables	2020	2021	2022	2023
First-line managers, N (% Response rate)	87 (49)	76 (32)	75 (34)	81 (37.9)
Woman N (%)	73 (83.9)	64 (84.2)	68 (90.7)	70 (87.5)
Man, N (%)	13 (14.9)	11 (14.5)	7 (9.3)	9 (11.3)
Missing value for gender, N	1	1	0	1
Age, mean + SD (years)	50.1 + 9.8	49.7 + 9.2	51.1 + 8.9	50.4 + 9.1
Total years as first-line manager + S.D. (years)	7.9 + 6.8	7.3 + 6.5	8.9 + 6.5	7.3 + 5.8
Years as first-line manager at current unit + S.D. (years)	4.6 + 4.9	4.3 + 5.1	4.8 + 4.5	4.2 + 4.1

SD = Standard deviation.

The decision to collect data annually during the autumn months was based both on practical and methodological considerations. An annual interval balanced feasibility, participant burden, and the ability to monitor trends over time. This approach aligns with recommendations in the methodological literature, such as those outlined by Wang and Cheng.^20^ This study followed the Strengthening the Reporting of Observational Studies in Epidemiology (STROBE) guidelines (https://www.strobe-statement.org/) to ensure transparency and methodological rigor.

### Questionnaires

The study's variables included expanded span of control, management and leadership performance, work-related stress, and job satisfaction. We used three established questionnaires and collected demographic data such as age, gender, total years of managerial experience, and years served as a first-line manager in the current unit, see Table 1.

The Ottawa Hospital Model of Nursing Clinical Practice and Clinical Management Span of Control Decision-Making Indicators tool (TOH-SOC)^21^ is a validated and comprehensive guide for assessing clinical managers’ expanded span of control. It consists of 21 items and evaluates a manager's span of control not only based on number of subordinates but also through three main decision-making categories: unit-focused, staff-focused, and program-focused, each with specific indicators. The unit-focused category considers unit complexity and material management. Unit complexity factors include hours of operation, unpredictability, patient turnover, risk of litigation, and adverse incidents. Material management is evaluated based on the time spent on work with equipment maintenance, purchasing, vendor interactions, and quality monitoring. The staff-focused category examines the number of people reporting directly to the clinical manager, the skill and autonomy of staff, staffing stability, and staff diversity. This includes volumes of staff reporting, percentage of novice nurses and nonprofessional staff, turnover rates, and absenteeism. Lastly, the program-focused category assesses the diversity of a unit through budgetary responsibilities. It considers factors such as the number of managers the respondent reports to, designated services, and the overall number of units managed. Each item response is assigned a level (Low = 1, Medium = 2, High = 3) indicated by a number and then multiplied by a specific weight (ranging from 2 to 5) defined in the manual, to calculate a weighted item score. These weighted scores are added together to produce a total score indicating the manager's expanded span of control. A higher total score reflects greater complexity or responsibility within the first-line manager role. The score rating guidelines state that a score of 0–60 suggests a span of control that is below acceptable and has growth potential. A score between 61 and 90 represents an appropriate span of control. If the score falls within the range 91–130, it indicates an excessive span of control, meaning that assistance may be needed.^21^

The Leadership and Management Inventory (LaMI-II)^22^ was used to evaluate first-line managers’ self-assessed leadership and management performance capacity. LaMI-II is a validated tool, and its 25 items focus on leadership and management perspectives. Of these, 17 items focus on leadership, assessing the first-line manager's ability to inspire, motivate, and develop their team, ensure effective communication, and foster a collaborative work environment. These items cover key leadership behaviors such as listening to employes, providing constructive feedback, being transparent with expectations, and managing conflicts effectively. Eight items are focused on management and operational responsibilities such as understanding the organization's structure, handling financial conditions, and ensuring compliance with laws and regulations. These items reflect a manager's capability to plan, organize, and oversee day-to-day activities, ensuring that their unit or department aligns with the broader organizational goals. The questionnaire uses a 5-point scale, ranging from “not at all” to “a very large extent.” The two factors, leadership and management, are scored 20–100 by adding up the item ratings, dividing by the maximum factor score, and then multiplying by 100. Cronbach's alpha in our 2020 sample showed values of .86 for leadership and .86 for management. Although no normative data are available for the LaMI-II, the mean scores for leadership and management ranged between 74.3 and 79.1 in a study by Skytt et al..^22^

The Health & Safety Executive Management Standards Indicator Tool (HSE),^23^ developed and published by the British Authority for Health Prevention and Safety at Work, was utilized to assess work-related stress among first-line managers. This questionnaire comprises 35 items and employs a 5-point scale and has confirmed acceptable validity and reliability.^24^ For 23 items, response options range from “never” to “always,” and for 12 items, response options range from “do not agree at all” to “completely agree.” The items are categorized into five standard work-related stressors: demands, control, relationships, role, and change. The sixth standard, support, is divided into two subscales: managers’ support and peers’ support. This section measures the feedback, assistance, and emotional support provided by managers, as well as the help and respect received from colleagues. The demands section evaluates various aspects of workload and work pressure, including conflicting demands, unattainable deadlines, heavy workload, neglecting tasks due to excessive workload, insufficient breaks, pressure to work long hours, fast work pace, and unrealistic time pressures. The control section assesses employes’ autonomy over their work, such as deciding when to take breaks, determining work speed, and having a say in completion of tasks. The relationships section focuses on workplace interactions, assessing exposure to personal harassment, bullying, friction, and strained relationships between colleagues. The role section measures clarity regarding job expectations, duties, and understanding how one's work fits the broader organizational goals. Lastly, the change section assesses the extent to which employes are consulted about changes at work and whether they understand how these changes will be implemented in practice.^23^ The HSE indicator tool is a widely used comprehensive instrument for examining work-related stress and psychological well-being across various occupational groups.^25–27^ The results of each standard can be categorized into one of four color-coded percentile ranges. These are Red (<20th percentile, urgent action required), Yellow (20th–50th percentile, improvement needed), Aqua (50th–80th percentile, good performance with potential for improvement), and Green (≥80th percentile, top performance to be maintained).^23^ Cronbach's alphas for demands, control, manager support, peer support, relationships, and role all showed acceptable values in our 2020 sample, ranging between .70 and .87. However, the subscale change showed a Cronbach's alpha value of .49 in the 2020 sample.

In order to assess job satisfaction among first-line managers, we used a single-item measure, asking the following question: “How satisfied were you with your work, considering your entire work situation?” The managers indicated their job satisfaction on a 7-point Likert scale. This single-item measure has demonstrated reliability and validity for assessing overall job satisfaction.^28,29^

### Statistical methods

We utilized descriptive statistics to outline the participant characteristics, as detailed in Table 1. To determine whether the data met the assumption of normality, a normality check was conducted. The results indicated varying degrees of normality across variables. Given the ordinal nature of collected data, the occurrence of non normal distribution, and the relatively small sample size, we opted to use non-parametric inferential statistics. To test H1, group comparisons were conducted using the Kruskal-Wallis test to assess potential differences across the years 2020–2023. This test helped determine whether the ratings related to job satisfaction, management support, peer support, and other factors varied significantly between the years.

For H2, a Spearman correlation analysis was performed to examine the relationships between job satisfaction and the variables included in the questionnaires for each respective year, as shown in Table 2.

**Table 2.** Variable descriptives and Kruskal-Wallis test.

Year	2020 (N = 87)		2021 (N = 76)		2022 (N = 75)		2023 (N = 80)		Kruskal-Wallis test	
Questionnaire and variable	Mean (SD) Median	95% CI	Mean (SD) Median	95% CI	Mean (SD) Median	95% CI	Mean (SD) Median	95% CI	H-value	P-value
TOH-SOC										
Expanded span of control	71.0 (8.9) 70.0	69.1–73.0	74.7 (9.9) 75.0	72.3–77.1	73.1 (8.9) 71.5	71.0–75.3	71.6 (10.0) 70.0	69.3–74.0	6.7	0.081
Missing values, n	3^a^		8^a^		7^a^		9^a^			
LaMI-II										
Leadership	83.5 (6.7) 82.9	82.0–85.0	82.8 (11.1) 82.4	81.4–84.2	83.0 (9.9) 82.3	74.7–79.3	84.3 (8.0) 83.5	82.5–86.1	2.0	0.572
Missing values, n	5^a^		1^a^		1^a^		-			
Management	76.6 (10.3) 78.8	74.4–78.8	75.7 (11.1) 77.5	73.2–78.3	77.0 (9.9) 77.5	74.7–79.3	77.9 (11.8) 77.5	75.3–80.6	2.1	0.561
Missing values, n	1^a^		1^a^		1^a^		-			
HSE										
Demands	3.0 (0.6) 3.1	2.9–3.2	3.0 (0.6) 3.0	2.9–3.1	2.9 (0.6) 2.9	2.7–3.0	2.9 (0.6) 2.9	2.8–2.9	5.1	0.164
Missing values, n	-		-		1^a^		-			
Control	3.5 (0.5) 3.7	3.4–3.7	3.5 (0.5) 3.5	3.4–3.6	3.6 (0.4) 3.7	3.5–3.7	3.6 (0.5) 3.7	3.5–3.7	3.5	0.327
Missing values, n	-		-		1^a^		-			
Manager's support	3.8 (0.8) 3.8	3.6–3.9	3.7 (0.8) 3.8	3.5–3.9	3.6 (0.8) 3.8	3.4–3.8	3.7 (0.9) 3.8	3.5–3.9	1.2	0.763
Missing values, n	-		-		-		-			
Peer support	3.8 (0.7) 4.0	3.7–4.0	3.8 (0.7) 3.8	3.6–3.9	3.8 (0.6) 4.0	3.7–4.0	3.9 (0.7) 4.0	3.7–4.0	1.6	0.664
Missing values, n	-		-		1^a^		-			
Relationship	2.0 (0.7) 2.0	1.9–2.2	2.0 (0.7) 2.0	1.9–2.2	1.9 (0.7) 1.8	1.8–2.1	2.1 (0.7) 2.0	1.9–2.2	1.9	0.591
Missing values, n	-		-		1^a^		-			
Role	4.0 (0.5) 4.0	3.9–4.1	4.0 (0.5) 4.0	3.9–4.1	4.1 (0.4) 4.0	4.0–4.2	4.2 (0.4) 4.2	4.1–4.3	1.6	0.186
Missing values, n	-		-		-		-			
Change	3.6 (0.6) 3.7	3.4–3.7	3.5 (0.6) 3.7	3.4–3.6	3.6 (0.5) 3.7	3.5–3.7	3.5 (0.6) 3.7	3.4–3.6	0.45	0.687
Missing values, n	-		-		1^a^		-			
Job satisfaction	5.0 (1.4) 5.0	4.7–5.3	4.8 (1.3) 5.0	4.5–5.1	5.0 (1.3) 5.0	4.8–5.4	5.0 (1.4) 5.0	4.7–5.3	1.9	0.593
Missing values, n	-		1^a^		-		-			

a Missing values.

SD = Standard deviation; TOH-SOC = The Ottawa Hospital Model of Nursing Clinical Practice and Clinical Management Span of Control Decision-Making Indicators tool; LaMI-II = Leadership and Management Inventory; HSE = Health & Safety Executive Management Standards Indicator Tool.

Correlation coefficients were calculated to assess the strength and direction of the relationships, aligning with the expected positive and negative correlations specified in the hypothesis. Correlation coefficients were calculated to assess both the strength and the direction of the relationships, allowing us to evaluate the expected correlations outlined in H2 across the cross-sectional data collected in 2020–2023. The correlation coefficient, r, measures the strength and direction of a linear relationship between two variables. The value of r can vary between −1 and 1. Cohen's^30^ guidelines categorize correlation effect sizes as follows: an r value between 0.10 and 0.29 is considered a small effect size, indicating a weak but noticeable correlation with potential practical significance. An r value between 0.30 and 0.49 is classified as a medium effect size, suggesting a moderate correlation. Lastly, an r value between 0.50 and 1.00 is considered a large effect size, signifying a strong correlation where the variables are strongly related. The significance of the correlations was determined using standard statistical criteria, with p-values indicating the level of significance.

In H3, we aimed to determine whether the correlations as outlined in H2 demonstrated comparable strength and consistency over time. The hypothesis assumed that the relationships between job satisfaction and factors such as expanded span of control, leadership and management performance and work-related stress would remain stable across the four years. By interpreting the strength of the correlations, we could evaluate whether the impact of these factors on job satisfaction fluctuated or was consistent.

Missing values were handled at the subscale/factor level. If the respective item response rate for a subscale/factor was greater than 50%, missing item values were replaced with the mean value for that item. Cases with more than 50% missing data for a specific subscale/factor in a questionnaire were excluded from further analysis. This approach is supported by Norman and Streiner,^31^ who describe mean imputation as a straightforward and unbiased method for replacing a missing value in a subscale or factor. The mean imputation could not be applied for the TOH-SOC questionnaire, as the questions have varying response options, and each indicator is crucial to the total score. Therefore, participants who did not respond to all questions in the TOH-SOC were excluded from the analysis. The number of missing values is reported in Table 2. We used IBM SPSS Statistics for Windows, Version 27.0 (IBM Corp, Armonk, New York) for the analysis.

## Results

The number of first-line managers participating varied over the years. Most participants were women, with a stable gender distribution across the years. A chi-square test confirmed no significant changes in gender proportion over time, indicating a consistent predominance of women in the sample. The mean age of the participants was stable, averaging around 50 years and ranging between 28 and 68 years. The total years of experience as a first-line manager averaged around 8 years, ranging between 0 and 35 years, and the years as a first-line manager at the current unit averaged around 4.5 years, ranging between 0 and 35 years. See Table 1 for an overview of the characteristics for each year.

The results for Hypothesis 1 indicated no significant differences in job satisfaction, management support, peer support, control, demands, relationships, role clarity, attitudes towards change, or leadership and management performance over the years (2020–2023), as determined by a series of Kruskal-Wallis tests. The detailed mean scores, standard deviations (SDs), confidence intervals (95% CIs), and Kruskal-Wallis test results are presented in Table 2. Job satisfaction among first-line managers remained consistent, with average scores ranging from 4.8 to 5.0 (H (3317) = 1.9, p = 0.593), indicating that the first-line managers were evenly satisfied with their job over time. The HSE variables, including demands, control, manager support, peer support, relationships, role, and change, consistently displayed similar mean values over time. The mean values for the control and change variables indicated good performance but showed potential for improvement. The mean values for control ranged from 3.5 to 3.6 (H(3317) = 3.5, p = 0.327), indicating that performance was consistently above average according to HSE questionnaire guidelines. However, there was room for improvement, as the scores did not reflect the highest level of performance. Similarly, the mean values for change ranged between 3.5 and 3.6 (H(3317) = 0.45, p = 0.930), indicating good performance with potential for improvement. It was evident that improvement was needed for demands, manager support, and peer support variables, when data were compared with norm values. The demands variable had a mean range of 2.9 to 3.0 (H(3317) = 5.1, p = 0.164). In two years of the cross-sectional survey, participants reported demands at a level indicating below-average performance requiring improvement, whereas in the other two years, their responses indicated an urgent need for action. Manager support was consistently scored around 3.7 to 3.9 (H(3317) = 1.2, p = 0.763), with three years showing above or average performance based on their responses and one year indicating top performance that should be maintained. Peer support had a mean that ranged from 3.8 to 3.9 (H(3317) = 1.6, p = 0.664), also showing three years of good performance with potential for improvement and one year of top performance that should be maintained.

Regarding the relationships and role variables, the results indicated that urgent action was needed. Relationship ratings fell into the range 1.8 to 2.1 (H(3317) = 12.3, p < 0.05). The role variable showed a mean range from 4.0 to 4.1 (H(3317) = 3.1, p = 0.378), with two years indicating that urgent action was needed and two years suggesting that improvement was needed.

In LaMI-II, leadership and management performance exhibited stable means, ranging from 82.8 to 84.3 (H(3311) = 2.0, p = 0.572) for leadership and from 75.7 to 77.9 (H(3315) = 2.1, p = 0.561) for management. The expanded span of control showed mean scores ranging from 71.0 to 74.7 (H(3291) = 6.7, p = 0.081) across the years. Based on the reference values, these scores indicate that the first-line managers had an appropriate expanded span of control. H1 was supported, as no significant differences were observed in job satisfaction and other related factors between 2020 and 2023.

In order to test H2, Spearman correlation analyses were performed to investigate the relationship between job satisfaction and other variables measured in the questionnaires for each year, as presented in Table 3. Here, we expected correlations between job satisfaction and specific variables such as expanded span of control, leadership and management performance, work-related stress, and workplace relationships – positive or negative depending on the variable. The expanded span of control (TOH-SOC) measure showed a mixed negative relationship with job satisfaction. However, this relationship was significant only in 2021 and 2023, with small to medium effect sizes. This highlights aspects of expanded span of control that may negatively impact job satisfaction. Leadership (LaMI-II) exhibited small positive correlations with job satisfaction, though these were only statistically significant in 2021 and 2023. The lack of significance in 2020 and 2022 indicates an inconsistent relationship, suggesting that although leadership may positively influence job satisfaction, its impact varies over time. The relationship between management (LaMI-II) and job satisfaction was positive, but only reached statistical significance in 2020 and 2022. This indicates that although management is generally associated with greater job satisfaction, its influence may be inconsistent. Thus, first-line managers’ self-assessed management practices are typically linked to greater job satisfaction, though their impact varies over time.

**Table 3.** Cross-sectional correlation with job satisfaction over the years.

Job satisfaction				
Variable	2020	2021	2022	2023
TOH-SOC				
Span of control	−0.054 (Not sig)	−0.289^a^	−0.182 (Not sig)	−0.334^b^
LaMI-II				
Leadership	−0.031 (Not sig)	0.261^a^	−0.014 (Not sig)	0.238^a^
Management	0.219^a^	0.212 (Not sig)	0.229^a^	0.177 (Not sig)
HSE				
Demands	−0.257^a^	−0.423^b^	−0.551^b^	−0.549^b^
Control	0.337^b^	0.479^b^	0.289^a^	0.530^b^
Manager support	0.318^b^	0.599^b^	0.533^b^	0.399^b^
Peer support	0.381^b^	0.515^b^	0.436^b^	0.311^b^
Relationship	−0.388^b^	−0.263^a^	−0.458^b^	−0.520^b^
Role	0.258^a^	0.231^a^	0.256^a^	0.384^b^
Change	0.328^b^	0.399^b^	0.231^a^	0.237^a^

Effect sizes according to Cohen^30^: Small: 0.10–0.29. Medium: 0.30–0.49. Large: 0.50–1.00.

^a^ Correlation is significant at the 0.05 level (2-tailed).

^b^ Correlation is significant at the 0.01 level (2-tailed).

TOH-SOC = The Ottawa Hospital Model of Nursing Clinical Practice and Clinical Management Span of Control Decision-Making Indicators tool; LaMI-II = Leadership and Management Inventory; HSE = Health & Safety Executive Management Standards Indicator Tool.

Over the four years, a consistent negative correlation was observed between demands and job satisfaction, with the strength of this negative relationship increasing over time. The correlation ranged from weak to strong. These findings suggest a significant link between higher job demands and lower job satisfaction, with the connection intensifying over time. In contrast, control exhibited a positive and significant correlation with job satisfaction over the years, with a small to large effect size. This implies that control over one's work is strongly associated with greater job satisfaction. The relationship became more pronounced over time. Manager support consistently demonstrated a positive correlation with job satisfaction, with a medium to large effect size. This suggests that first-line managers perceiving support from their managers reported significantly greater job satisfaction.

Similarly, peer support showed a positive and significant correlation with job satisfaction across the years, with a medium to large effect size, underscoring the substantial contribution of supportive colleagues to greater job satisfaction. Conversely, the subscale relationships (including, e.g. bullying and strained relationships) showed a negative correlation with job satisfaction, with the negative impact increasing over time. Although the relationship in 2021 was weak, it was still significant, and effect sizes ranged from small to large across the years. The growing strength of this correlation highlights a strong association between poor workplace relationships and lower job satisfaction. Role exhibited a positive correlation with job satisfaction, with the negative effect size ranging from small to large. The relationship grew stronger over time. This highlights the importance of clarity and understanding of the first-line manager's role within the organization. Change generally displayed a positive correlation with job satisfaction, with a small to medium effect size, suggesting a moderate association between effective change management and greater job satisfaction. Given the mixed results, H2 was partially supported.

H3 proposed that the correlations between job satisfaction and expanded span of control, leadership and management performance, and work-related stress would remain consistent from 2020 to 2023. However, the results did not fully support this hypothesis. Several variables, such as expanded span of control and leadership, fluctuated over the years. Work-related stress maintained a consistent negative relationship with job satisfaction, but other factors showed variability. H3 was rejected, as the correlations did not remain stable over time; see Table 3 for details.

## Discussion

This study involved a repeated cross-sectional analysis to explore factors, including expanded span of control, leadership and management performance, work-related stress, and job satisfaction among first-line managers in a Swedish healthcare organization from 2020 to 2023. H1, which proposed no significant changes in these variables over the four years, was supported. This suggests that these aspects of the first-line managers’ roles remained relatively stable despite the dynamic challenges faced by the healthcare sector, such as COVID-19. However, it is important to consider that underlying stressors and challenges still may have intensified during the pandemic, as suggested by previous studies. For instance, Björk et al.^14^ reported heightened pressures on first-line managers during COVID-19, and Gilbert et al.^1^ highlight the multifaceted demands impacting healthcare managers’ work environments. These findings underscore the complexity of the organizational context, suggesting that while certain measured outcomes appeared stable, the experiences of first-line managers might be more nuanced.

Work-related stress, especially related to demands and workplace relationships, emerged as a critical factor negatively affecting job satisfaction. This is consistent with the literature, which shows that healthcare managers often face higher stress levels due to frequent political reforms, organizational changes, and increasing workloads.^2^ Wynen et al.^3^ highlighted that frequent organizational changes amplify stress and disrupt workplace dynamics, which aligns with our findings regarding workplace relationships. Jäppinen et al.^12^ further underscore that high workloads, combined with role ambiguity, contribute to elevated stress levels among healthcare managers. The demanding nature of first-line management requires the ability to make rapid decisions while simultaneously managing heavy workloads. As our results indicate, this often exacerbates stress and reduces job satisfaction. These challenges are further emphasized by Moore et al.,^6^ who expressed the need for additional resources and support to alleviate such pressures. Leadership and management performance showed inconsistent relationships with job satisfaction, which were influenced by external stressors. Organizational support serves a crucial role in buffering against stress and sustaining job satisfaction. Without addressing chronic stressors such as high demands and unclear roles, managers remain at risk of burnout and turnover, even if overall job satisfaction appears stable.^15,16^

In line with the findings of Labrague et al.,^13^ stressors such as high job demands, resource limitations, and inadequate staffing levels were consistently associated with lower job satisfaction. Similarly, Jankelová et al.^32^ emphasized the importance of perceived organizational support and feedback-seeking behavior in mitigating work-related stress for first-line managers. Their study indicated that when managers feel supported by their organization and actively seek feedback, they gain clarity and guidance, which helps them manage stress more effectively. Addressing these stressors through improved organizational support and workload management could help prevent burnout and reduce turnover intentions among first-line managers.^13^

H2 posited positive correlations between job satisfaction and leadership and management performance, managerial support, peer support, control, role, and change, and negative correlations with demands, relationships, and expanded span of control. The findings from this study provide partial support for this hypothesis. Expanded span of control exhibited a mixed, negative relationship with job satisfaction, indicating that managing more complex responsibilities can detract from overall satisfaction.^11^ Although expanded span of control remained relatively stable throughout the study, the mixed results regarding its relationship with job satisfaction suggest that managing more complex responsibilities and diverse tasks can create challenges for first-line managers. A greater span of control can make it difficult for a first-line manager to maintain close relationships with their employes, negatively affecting their leadership effectiveness.^9^ Previous research in the Swedish municipal healthcare sector has indicated that first-line managers often supervise larger teams than in other municipal sectors, such as technical services.^33^ Supervising a large team can exacerbate stress and reduce job satisfaction. Furthermore, research emphasizes that first-line managers in female-dominated municipal welfare sectors, such as healthcare, oversee larger teams and receive less organizational support than their counterparts in male-dominated sectors, contributing to increased stress levels. This imbalance affects first-line managers’ abilities to maintain effective leadership and manage the work environment.^34^

Although leadership and management performance demonstrated positive – albeit inconsistent – associations with job satisfaction, these factors appeared secondary to the impact of work-related stress. This highlights that though assessments of leadership and management performance are important, the persistent stressors exacerbated by limited decision-making authority and unclear role boundaries impact first-line managers’ job satisfaction more. Additionally, a study by Hagerman et al.^35^ showed that first-line managers in municipalities with larger teams (≥31 subordinates) experienced more challenges due to limited resources and support, which negatively impacted their leadership capabilities. However, these managers demonstrated slightly higher leadership and management performance than those with smaller teams, although the difference was not statistically significant. This finding aligns with our results, where expanded span of control showed a mixed impact on job satisfaction, suggesting that although managing larger teams can be challenging, it may also foster stronger leadership skills when adequate organizational support is present. In contrast, a study by Wong et al.^36^ found that first-line managers in Canadian healthcare oversaw an average of 79.8 subordinates.

H3 suggested that the connections between job satisfaction and expanded span of control, leadership and management performance, and work-related stress would remain consistent from 2020 to 2023. However, the results showed variability, especially in the impact of span of control and leadership performance on job satisfaction. This aligns with a systematic review by Penconek et al.,^17^ which emphasized that factors influencing job satisfaction, such as leadership and organizational characteristics, are often dynamic and subject to change due to evolving workplace environments. On the other hand, work-related stress had a negative impact on job satisfaction throughout the study period, a result strongly supported in the review by Penconek et al.,^17^ which found that high stress levels are consistently linked to decreased job satisfaction among first-line managers.

The subscale change within the HSE assesses how first-line managers perceive changes, focusing on aspects such as being informed about changes and feeling involved in the change process. However, this subscale demonstrated low internal consistency (Cronbach's alpha = .49), indicating that the items in the subscale do not consistently measure the concept of “change.” This low reliability suggests potential issues with how well the questions capture the complexities of change in the workplace. Change processes are often multifaceted, involving various dimensions that may be difficult to encapsulate within a limited number of items. According to Tavakol and Dennick,^37^ reliability is influenced by how well items measure a single concept. A short scale, like the change subscale, may tend to have low alpha values. The low Cronbach's alpha value highlights the need for further refinement or expansion of this subscale, to more accurately capture the various facets of change-related stress and its impact on first-line managers.

One of the positive outcomes identified in this study was the consistent influence of managerial support and control on job satisfaction across the study period. Despite the various challenges faced by first-line managers, the correlation between these factors and job satisfaction remained strong. Both managerial support and the sense of control over one's work showed medium to large effect sizes, underscoring their importance as key resources that buffer against the negative impact of work-related stress. The findings in our study align well with the theoretical framework provided by the JD-R model.^38^ According to the JD-R model, job demands – such as heavy workloads and interpersonal conflicts – can lead to stress, burnout, and reduced job satisfaction. However, job resources such as autonomy, managerial support, and role clarity can mitigate these negative effects. Our results showed that demands, workloads, and relationships had a strong, negative impact on job satisfaction. The results also highlighted the positive influence of resources like control, manager support, and peer support, which were consistently linked to greater job satisfaction. As control and support increased, so did job satisfaction, reinforcing the idea that resources play a crucial role in mitigating the negative effects of job demands. Furthermore, our findings align with the evolving JD-R model,^18^ which emphasizes the dynamic interaction between demands and resources. Bakker and Demerouti^18^ propose that job resources not only buffer the impact of job demands but also act as motivators that enhance job performance and well-being. Our results support this expanded understanding, demonstrating how key resources can alleviate stress and foster greater job satisfaction.

The subscale relationships revealed a particularly strong and consistent negative impact on job satisfaction across all study years. Moreover, poor communication, lack of support from superiors and peers, and team conflicts were significant stressors. Previous research by Arakelian et al.^39^ similarly found that unresolved conflicts and inadequate communication can lead to feelings of isolation and disrespect, contributing to job dissatisfaction. Organizations should focus on fostering better communication and building supportive relationships to improve job satisfaction among first-line managers. Although our study did not focus on the COVID-19 pandemic, its impact on first-line managers cannot be overlooked. The pandemic intensified pressures, underscoring the need for effective stress management to support job satisfaction in crisis conditions.^14^ An integrative review by Aydogdu^40^ showed that first-line managers experienced severe physical and psychological burdens throughout the pandemic, increasing stress and dissatisfaction. Despite this, our data showed no significant change in expanded span of control, leadership and management, work-related stress, or job satisfaction. This suggests that first-line managers’ job satisfaction was not adversely affected, despite the increased pressures. The stability can be attributed to effective organizational support structures and the flexibility and responsibility displayed by first-line managers in solving the problems created by the pandemic. Our results emphasize the importance of maintaining strong support systems to ensure the well-being and effectiveness of first-line managers, both in times of crisis and under normal conditions.

### Limitations

Several limitations must be considered. Low response rates (32% to 49%) may have introduced response bias. Stressed first-line managers may have been less likely to participate, potentially leading to underestimation of work-related stress. This aligns with a systematic review by Blumenberg and Barros,^41^ who found that web-based surveys often have lower response rates. Moreover, recall bias may have influenced the self-reported data, as participants’ ability to accurately recall past experiences from up to a year ago may vary.

The cross-sectional design limitscausal inferences, as it captures data at specific points in time rather than tracking changes longitudinally. Cross-sectional studies are inherently limited in their ability to establish causality between variables, as exposure and outcome are measured simultaneously, making it challenging to determine temporal relationships.^20^ The use of convenience sampling may introduce selection bias, limiting generalizability,^20^ especially as the study focused on a single Swedish region. Moreover, the study does not account for certain personal or organizational factors that could influence job satisfaction. For instance, variables like organizational commitment and individual attributes including marital status, or education level might have provided deeper insights into the interplay between job satisfaction and managerial roles. In this study, missing data remained below 5% for all variables, except for one subscale Leadership in LaMI-II, where missing data reached 5.7%. While this remains within the acceptable threshold identified by Norman and Streiner,^31^ it is important to acknowledge the potential for slight underestimation of variance. The study's limitations may affect how we interpret the relationships between expanded span of control, leadership and management performance, work-related stress, and job satisfaction. Despite these limitations, the findings showed stability in key factors, even during the COVID-19 pandemic. This could affect the generalizability of the findings, mainly if those who chose to participate differed systematically from those who did not. To overcome this limitation, a national cohort study could be conducted, following first-line managers across multiple regions and organizations over time. However, this approach might introduce increased variability due to differences in organizational structures, resources, and working conditions, which could affect the ability to draw conclusions.

### Implications and future research

The findings from this study underscore the importance of organizational support and resource allocation in enhancing job satisfaction among first-line healthcare managers. High demands and strained workplace relationships were found to significantly diminish satisfaction, whereas support from peers and higher management, coupled with greater control over work, markedly improved it. To address these challenges, organizations should prioritize strategies such as reducing workloads, fostering positive workplace relationships, and clarifying the roles and responsibilities of first-line managers. Initiatives like structured mentorship programs and transparent communication can be effective in mitigating stress, enhancing managerial confidence, and ultimately promoting greater job satisfaction. Future research should explore the long-term effects of interventions aimed at reducing stress and improving support for first-line managers. Additionally, investigating personal and organizational factors, such as individual resilience and leadership styles, could provide further insights into enhancing managerial well-being and effectiveness.

## Conclusion

Work-related stress, driven particularly by demands and workplace relationships, emerged as a critical factor that consistently reduced job satisfaction among first-line managers. Reducing workloads, clarifying role expectations, and fostering better relationships through enhanced support structures could significantly improve job satisfaction and managerial effectiveness. If organizations address these factors, they can potentially enhance the work environment for first-line managers.
